# De Novo Assembly, Characterization and Comparative Transcriptome Analysis of the Mature Gonads in *Megalobrama terminalis*

**DOI:** 10.3390/ani15152184

**Published:** 2025-07-24

**Authors:** Yicheng Zhou, Weiqian Liang, Kaifeng Wang, Peng Zheng, Shengyue Lin, Haiying Yang, Guojun Cai, Ziyan Deng, Chong Han, Qiang Li

**Affiliations:** School of Life Sciences, Guangzhou University, Guangzhou 510006, China; ztc1756822657@163.com (Y.Z.); liangweiqian1112@163.com (W.L.); 13530626652@163.com (K.W.); zp3209195542@163.com (P.Z.); 13923505936@163.com (S.L.); 13825355938@163.com (H.Y.); 17875695995@163.com (G.C.); 18813619597@163.com (Z.D.)

**Keywords:** *Megalobrama terminalis*, de novo assembly, gonads, DEGs

## Abstract

*Megalobrama terminalis* is a significant aquatic fish in South China, renowned for its tasty meat. Nonetheless, related studies are deficient concerning the gonadal development of *M. terminalis*. This paper presents the first comparative transcriptome analysis of the gonads of female and male *M. terminalis*. A total of 84,886 unigenes were assembled, with 42,322 effectively annotated to the Nr, SwissProt, KEGG, KOG, and GO databases. Furthermore, comparative transcriptomic analysis of *M. terminalis* was conducted to examine its gonadal development. A total of 14,972 differentially expressed genes (DEGs) were discovered. In the testis, the expression of 11,928 unigenes was significantly upregulated, while 3044 were significantly downregulated. Numerous DEGs associated with steroidogenesis, gonadal differentiation and development, and gametogenesis in teleost fish were identified. The results provide empirical support for further study of genes and pathways associated with sex determination and gonadal differentiation in teleost fish.

## Simple Summary

*Megalobrama terminalis* is an economically important fish indigenous to South China. However, there is less research on gonadal differentiation and development in *M. terminalis*. In this study, the gonadal transcriptome of female and male *M. terminalis* was analyzed for the first time. Many differentially expressed genes (DEGs) relevant to steroidogenesis, gonadal differentiation and development, and gametogenesis were identified after comparing the transcriptomes of male and female gonads. The annotation results of DEGs showed that some reported genes related to gonadal differentiation in teleost fishes may play essential roles in *M. terminalis*. Results of this study will provide data to support the reproduction and breeding of *M. terminalis*.

## 1. Introduction

*Megalobrama* are widely distributed freshwater fish in China, inhabiting the Yangtze River, Pearl River, Yellow River, and Hainan Island, among other river systems. They are favored by the public for their tender texture and exceptional taste, which makes them an important aquatic product in China [1,2,3]. Therefore, genomics [4], transcriptomics [5], molecular markers [6], and germplasm survey of this genus have been extensively investigated. However, research on *M. terminalis*, a species of *Megalobrama*, is limited.

*M. terminalis* belongs to *Megalobrama*, Culterinae, Cyprinidae, Cypriniformes, mainly distributed in the Pearl River system and Hainan Island water system, and it is an economically important fish endemic to the South China region [7,8]. Due to the superimposed effects of multiple anthropogenic factors such as hydraulic engineering, channel dredging, water pollution, and overfishing, *M. terminalis* resources have been decreasing since 1970, and *M. terminalis* resources dropped to roughly 5 tons in the 1980s [9,10]. A few studies have been conducted on *M. terminalis*, mainly focusing on the taxonomic status and evolutionary history through mitochondrial sequences [11,12,13], exploration and improvement of the feed composition [14,15], and artificial propagation and crossbreeding techniques for *M. terminalis* [16,17]. However, less attention has been paid to the gonadal development, differentiation, maturation, and gametogenesis of *M. terminalis*. To better promote the study of the mechanisms of gonadal development and reproduction of *M. terminalis*, it is vital to explore the genes and pathways related to gonadal differentiation using the RNA-seq technique.

Fish are different from higher vertebrates. Their sex determination and gonadal differentiation are intricate and are affected by environmental and genetic factors, and many other variables. Up to now, some important pathways and genes have been identified in fish [18,19]. Moreover, due to the diversity of fish species and living habitats, gonadal development and sex differentiation of fish are diversified [20]. Next-generation sequencing (NGS)-based transcriptome sequencing can quickly and cost-effectively generate large amounts of transcript sequences and mRNA expression data with high-throughput advantages, enabling efficient gene expression profiling and providing data support for fish research [21]. Currently, NGS-based transcriptome sequencing has been applied in many fish species such as *Coreoperca whiteheadi* [22], *Scortum barcoo* [23], *Siganus oramin* [24], and *Spinibarbus hollandi* [25]. These studies provide important insights into the mechanisms of gonadal development, reproduction-related genes, and complement the gaps in gonadal transcriptome studies in specific fish species.

In the present study, gonadal RNA-seq of female and male *M. terminalis* was performed using the Illumina platform, followed by de novo assembly and gene annotation. The comparative transcriptomics approach was employed to systematically analyze gene expression differences potentially involved in gonadal differentiation and gametogenesis. This study aims to further expand the existing genetic and genomic data resources, and through deepening gene expression analysis and functional gene mining, to screen and identify genes related to key reproductive processes such as gonadal differentiation and development and gametogenesis as comprehensively as possible, to lay a solid data foundation for subsequent in-depth investigation of the molecular mechanism of reproduction in *M. terminalis*.

## 2. Materials and Methods

### 2.1. Sampling

Six 2-year-old and sexually mature *M. terminalis* individuals (three females and three males) were acquired from an aquaculture farm in Shaoguan, Guangdong province. The fish were subsequently anesthetized and euthanized using MS-222 (STAHERB, Changsha, China) [26]. Gonadal tissues were immediately dissected, placed in an RNA keeper (Vazyme, Nanjing, China), and stored at −20 °C.

### 2.2. RNA Extraction and cDNA Library Construction

Total RNA from gonadal tissues was extracted using FreeZol regent (Vazyme, Nanjing, China), which was operated strictly according to the manufacturer’s instructions. RNA concentration and purity were initially checked using a NanoPhotometer N60 (Implen, Munich, Germany), and further assessed for integrity and quantitative analysis by an Agilent 2100 Bioanalyzer (Agilent Technologies, Santa Clara, CA, USA). Eukaryotic transcriptome sequencing libraries were constructed using the NEBNext^®^ Ultra™ RNA Library Preparation Kit (NEB, Ipswich, MA, USA), which includes the following steps: enrichment of mRNAs with polyA tails using Oligo (dT) magnetic beads, fragmentation of mRNAs by ultrasound; synthesis of mRNAs in an M-MuLV reverse transcriptase system using fragmented mRNAs and random oligonucleotides as primers. The first strand of cDNA was subsequently synthesized in the DNA polymerase I system using dNTPs as the raw material for the second strand. The purified double-stranded cDNA was end-repaired, added A tails, and ligated to the sequencing adaptor. cDNAs of about 200 bp were selected with AMPure XP beads (Beckman Coulter, Brea, CA, USA), PCR amplification was performed, and the PCR products were purified again using AMPure XP beads, and the libraries were finally obtained.

### 2.3. Library Sequencing, De Novo Assembly, and Annotation

Qualified libraries underwent a pair-end 150 sequencing strategy (PE150) utilizing the Illumina Novaseq6000 sequencing platform (Illumina, San Diego, CA, USA), yielding a minimum sequencing data volume of 6 Gb per sample. Raw sequencing data were filtered to remove reads with adapter contamination and low quality (N base >10%) to get clean reads, which were subsequently stored in FASTQ format. De novo assembly of the transcriptome was performed using Trinity 2.8.4 [27], and the quality was evaluated using N50 and BUSCO 3.0.2 [28]. At last, sequences of coding regions of the assembled unigene were predicted by six reading frames (3 forward and 3 reverse).

The predicted protein sequences were aligned with the Nr database (https://ftp.ncbi.nlm.nih.gov/blast/db/ (accessed on 20 April 2025)) and the SwissProt database (https://www.uniprot.org/ (accessed on 2 May 2025)), selecting the coding mode of the optimal alignment (maximum alignment score) as the gene’s coding region. Homology predictions of unigenes were subsequently done through comparisons with the Nr, KOG (https://www.ncbi.nlm.nih.gov/COG/ (accessed on 3 May 2025)), KEGG (http://www.kegg.jp, (accessed on 4 May 2025)), and SwissProt databases using the BLAST+ 2.6.0. The Nr results were subsequently annotated using Blast2GO 6.0 (GO, http://eggnog5.embl.de/download/emapperdb-5.0.2/ (accessed on 13 May 2025). Unigenes were categorized based on biological processes, cellular components, and molecular functions.

### 2.4. Identification of Differentially Expressed Genes and Enrichment Analysis

Clean reads from each sample were aligned to the assembled transcripts utilizing hisat2 [29], and gene expression metrics, including Fragments Per Kilobase of exon model per Million mapped fragments (FPKM) and coverage, were computed using RSEM 1.2.19 [30]. The python script prepDE.py was used to convert the output of stringtie to the compatible format of the edgeR package (V3.6), and edgeR was used for the analysis of differentially expressed genes (DEGs), aiming to identify the significantly differentially expressed genes with the filtering thresholds of FDR value < 0.05 and |log2FC| > 2. Finally, using the clusterProfiler program in the R package, significant GO terms and KEGG pathways (FDR < 0.05) were further enriched based on these DEGs following Fisher’s exact test and Benjamini-Hochberg correction [31].

### 2.5. Validation of DEGs Using Quantitative Real-Time PCR

The reliability of the data was confirmed through quantitative reverse transcription polymerase chain reaction (qRT-PCR). Sixteen DEGs related to gonadal differentiation and development were chosen for validation. *β-actin* was selected as the reference gene. Primers were designed using Primer-BLAST (Table 1). The specific steps were as follows: cDNA template was synthesized by HiScript III RT SuperMix (Vazyme, Nanjing, China), and amplified by SYBR Green qPCR Mix (GDSBIO, Guangzhou, China) on the LightCycler 480 platform. The reaction program was as follows: The reaction program included pre-denaturation at 95 °C for 3 min, followed by 40 cycles of 95 °C for 10 s, 60 °C for 20 s, and 72 °C for 20 s, concluding with a final extension at 72 °C for 5 min, which included melting curve analysis. Each sample was subjected to three biological replicates, and the results were normalized using the comparative CT method (2^−∆∆CT^) [32]. Results are presented as the mean ± standard error of the mean (Mean ± SEM).

### 2.6. Gonadal Histology

Gonadal tissues were first dehydrated in 70–100% alcohol in a gradient (gradient of 10%) and then made transparent with xylene. And then embedded in paraffin for 3 h before cutting into slices ranging from 3 to 6 μm. Qualified slices were dried overnight at 42 °C and stained with hematoxylin and eosin. The transparent slides were promptly sealed using neutral resin and left to air-dry at 25 °C overnight. Finally, slices were observed and photographed using a light microscope (Leica DM3000, Wetzlar, Germany) [23].

## 3. Results

### 3.1. Overview of Transcriptome Assembly Quality

**Figure 1:**
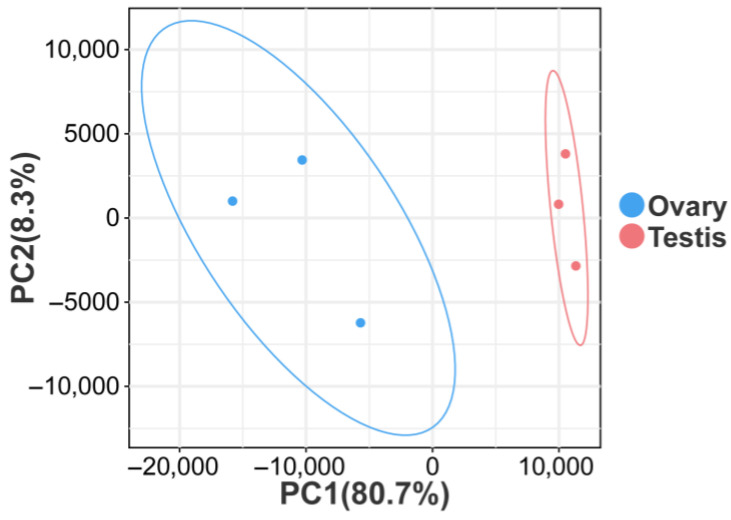
PCA of six samples.

**Figure 2:**
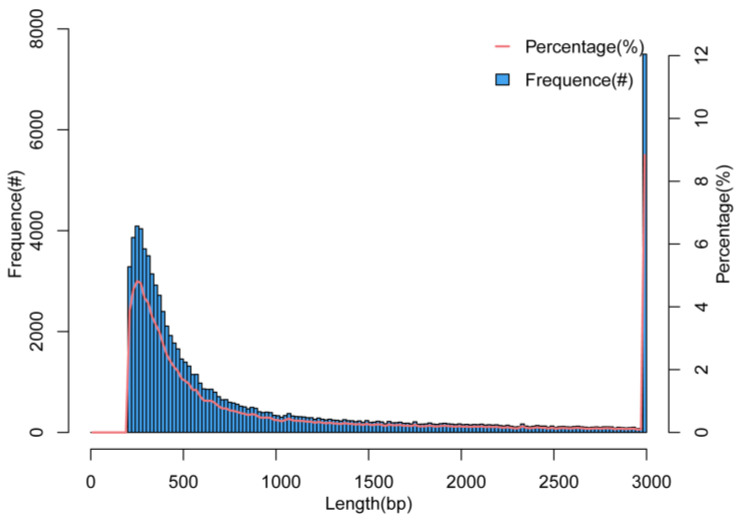
Length distribution of all assembled unigenes of the *M. terminalis* gonadal transcriptome. The “#” refers to numbers.

cDNA libraries were constructed from three ovary and three testis samples using RNA-seq. Following data quality control and filtering of low-quality data, clean reads totaling 41.18 GB were generated, with an average of 6.86 GB per sample, utilizing the Illumina HiSeq platform. Base quality values Q20 and Q30 were 99.28% and 97.36%, respectively (Table 2). In addition, PCA results showed significant differences between testis and ovary samples and good consistencies in parallel samples (Figure 1). A total of 84,886 unigenes were successfully assembled, with an overall length of 92.26 MB. The lengths of individual unigenes varied from 201 to 20,142 bp, with an average length of 1086 bp and a N50 of 2238 bp (Table 3). However, the majority of unigenes were 1–1000 bp in length (70.7%) while those with lengths greater than 1000 accounted for only 29.3% of the total (Figure 2).

### 3.2. Unigene Annotation

**Figure 3:**
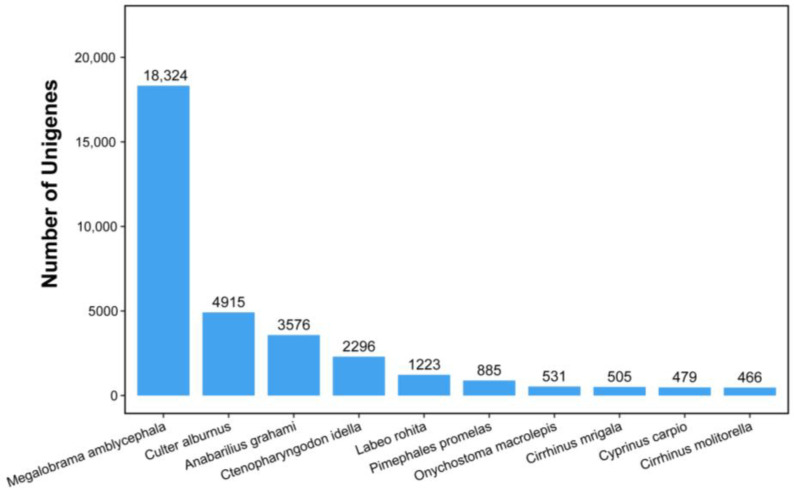
The species distribution of the results of the Nr annotation.

A total of 42,322 unigenes were successfully annotated among all the assembled unigenes. The number of annotated unigenes and their percentage in the five major databases (Nr, SwissProt, KEGG, GO, and KOG) in descending order were 40,529, 95.8%; 25,877, 61.1%; 25,801, 60.9%; 25,055, 59.2%; and 20,139, 47.6%, respectively (Table 3). In the Nr annotation, the results showed that *Megalobrama amblycephala* had the largest number of homologous genes with *M. terminalis* (18,324), followed by *Culter albumus* (4915), while the remaining species had less than 4000 homologous genes (Figure 3).

**Figure 4:**
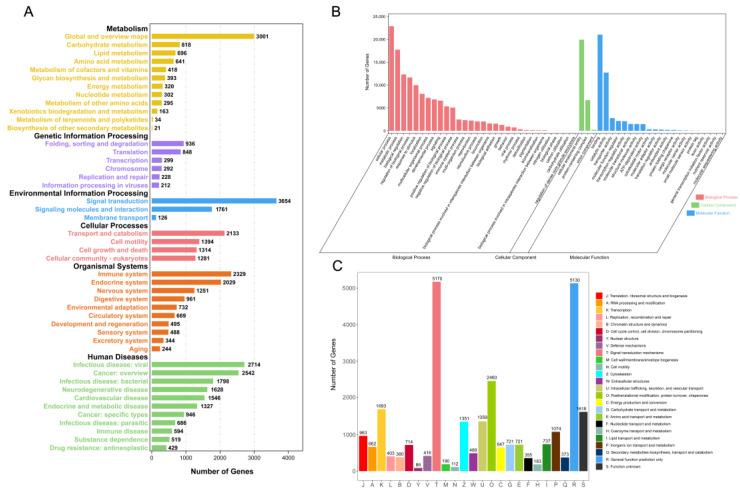
Gene function classification based on KEGG (**A**), GO (**B**), and KOG (**C**).

In addition, all unigenes were further annotated in KEGG, GO, and KOG databases to predict their functions and categorize them. The KEGG database categorized 25,801 unigenes into metabolism, genetic information processing, environmental information processing, cellular processing and organismal systems (Figure 4A); In the GO database, a total of 25,055 unigenes were annotated into three functional categories of biological process, cellular component and molecular function and the most representative terms in each category were cellular process, cellular anatomical entity and binding (Figure 4B); Finally, the KOG database categorized 20,139 unigenes into 25 families, of which the two families with the highest proportion were signal transduction mechanisms and general function prediction only (Figure 4C). More results of KEGG and GO annotation can be found in Tables S1 and S2.

### 3.3. Differential Expression Analysis

**Figure 5:**
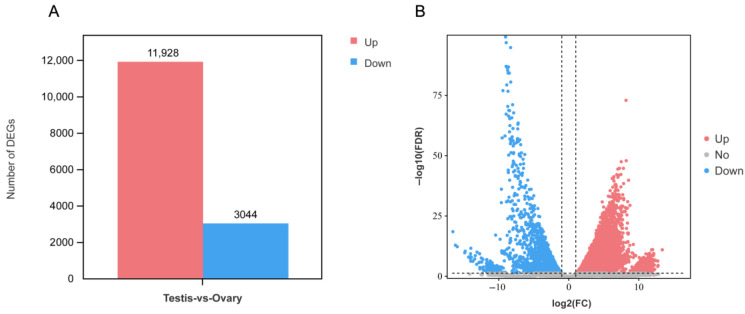
DEGs in testis and ovary samples. (**A**) Number of DEGs. (**B**) Volcano plot of DEGs. The red, blue, and gray dots, respectively, represent significantly upregulated genes, significantly downregulated genes, and genes without significant expression difference in testes.

**Figure 6:**
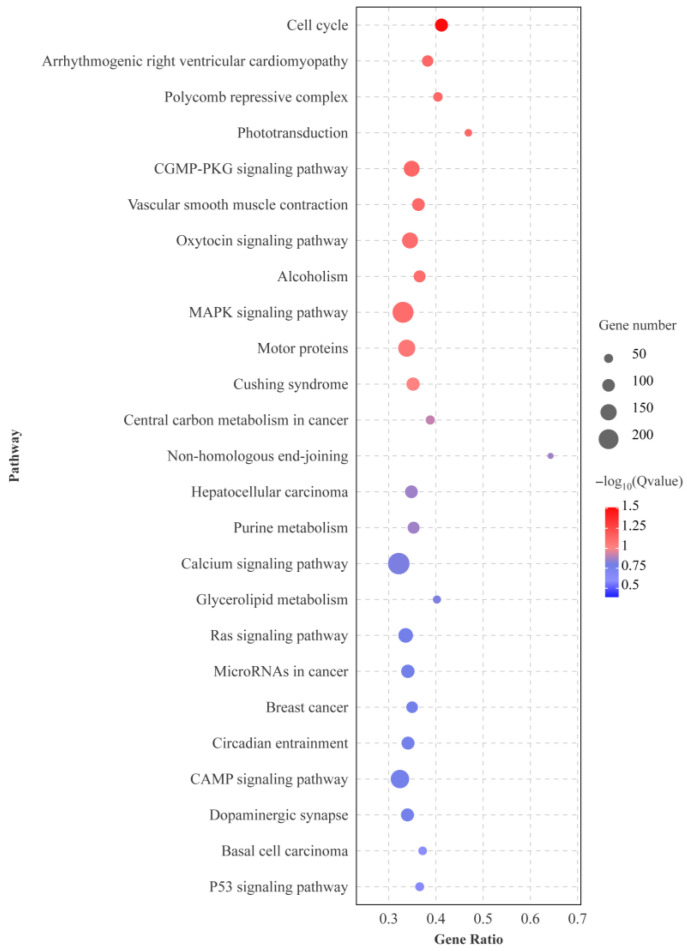
Top 25 KEGG enrichment analysis. The horizontal axis is the ratio of the number of differential genes annotated to the KEGG pathway to the total number of differential genes. The vertical is the enriched KEGG pathways. The size of the dots represents the number of genes annotated on the KEGG pathway term. The color key, from red to blue, represents significant enrichment.

A total of 14,972 DEGs were identified between ovary and testis samples, of which 11,928 were significantly upregulated in the testis and 3044 were significantly downregulated (Figure 5). KEGG enrichment analysis showed that among the top 25 significantly enriched pathways, there are multiple pathways related to gonad development, such as cell cycle, MAPK signaling pathway, and Calcium signaling pathway (Figure 6).

In addition, several DEGs related to steroidogenesis, gonadal differentiation and development, and gametogenesis were identified. Aromatase (*cyp19a1a*), cytochrome P450 26A1 (*cyp26a1*), estradiol 17-beta-dehydrogenase 1 (*hsd17b1*) and synaptonemal complex protein 2-like (*sycp2l*) are predominantly expressed in ovary; Doublesex- and mab-3-related transcription factor 1 (*dnmrt1*), transcription factor SOX-30-like (*sox30*), tektin-4 (*tekt4*), and cholesterol 25-hydroxylase-like protein (*ch25h*) are predominantly expressed in testis (Table 4).

### 3.4. Validation of Transcriptomic Data by qRT-PCR

**Figure 7:**
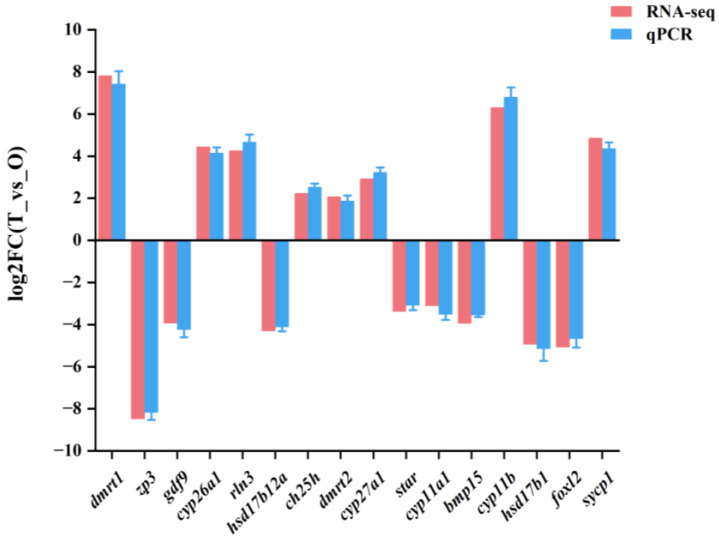
Validation of transcriptomic data by qRT-PCR. T: testis; O: ovary.

The differential expression results of qRT-PCR, including 16 DEGs (*dmrt1*, *cyp11b*, *sycp1*, *cyp26a1*, *rln3*, *cyp27a1*, *star*, *gdf9*, *bmp15*, *hsd17b12a*, *hsd17b1*, *foxl2*, and *zp3*), were consistent with those of RNA-seq (Figure 7, Table 4).

### 3.5. Gonadal Histology Analysis

**Figure 8:**
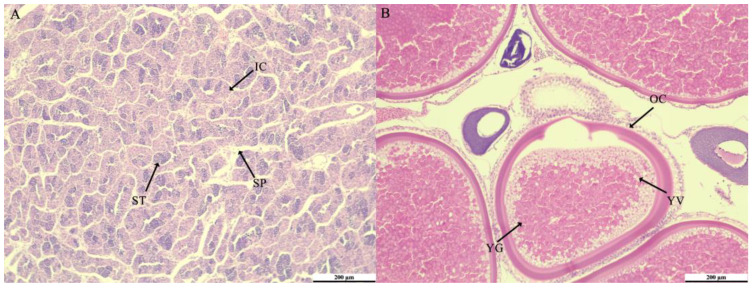
Testis (**A**) and ovary (**B**) histology. IC: Interstitial cells; ST: spermatid; SP: sperm; OC: Oocyte; YG: Yolk granules; YV: Yolk vacuoles.

The testes and ovaries of the two-year-old *M. terminalis* were utilized for histological analysis. The testes had numerous spermatids and interstitial cells, coupled with a limited quantity of sperm (Figure 8A), whereas the ovaries were abundant in mature oocytes (Figure 8B). Histological results showed that the gonads of male and female *M. terminalis* were mature.

## 4. Discussion

Sex hormone synthesis, gonadal differentiation, and gametogenesis are complex processes involving numerous specific pathways and genes. Transcriptome sequencing has been widely used to obtain comprehensive genetic information from specific organs and tissues of organisms, as it effectively identifies differences in gene expression among different treatment groups. *M. terminalis* is a commercially significant fish in southeastern China. However, overfishing has resulted in a substantial decline of its wild resources in recent years, necessitating urgent research on its captive breeding and cultivation. In this study, genes related to gonadal differentiation and gametogenesis in *M. terminalis* were revealed by transcriptome sequencing for the first time.

### 4.1. DEGs Involved in Steroidogenesis

Sex hormones are classified as steroid hormones, essential for embryonic development, gonadal differentiation, and gametogenesis in teleost fish [33]. Steroids are synthesized from their precursor, cholesterol. Notably, within our annotated genes, *cyp27a1* is involved in the initial hydroxylation reaction of cholesterol in the Japanese Lamprey *Lethenteron reissneri* [34], while *ch25h*, which is crucial for cholesterol metabolism, has been reported to exhibit high expression levels in the gonads of *Cynoglossus semilaevis* [35]. In addition, *cyp11a1* encodes the type I cytochrome P450 enzyme located in mitochondria, which catalyzes the transformation of cholesterol into pregnenolone [36]. *star* encodes the steroidogenic acute regulatory protein (StAR), which facilitates the transport of cholesterol from the mitochondria and has been reported to regulate the transformation of cholesterol to steroids in some teleost fish [37,38].

11-Ketotestosterone (11-KT) and 17-estradiol (E2) are the major androgens and estrogens in teleost fish [25]. Their production requires the involvement of several P450 family and HSD family genes [39], and we identified several related genes. Previous studies have shown that *hsd17b12a* catalyzes the transformation of 11-ketoandrostenedione (11-KA4) to 11-KT in the testis of *Anguilla japonica* [40]. Additionally, *hsd17b1* and *hsd17b12a* were predicted to participate in the synthesis of E2 in the ovaries of female Japanese eels [41]. The CYP11B enzyme facilitates the conversion of progesterone to the steroid testosterone and androstenedione in humans [42]. Concurrently, *cyp11b* was identified as being expressed in the ovaries and testes of *Paralichthys olivaceus*, and a reduction in the transcript level of *cyp11b* following E2 treatment implies its potential role in estrogen synthesis in fish [43]. Additionally, the deletion of *cyp17a1* resulted in decreased E2 levels in zebrafish plasma, while concurrently increasing progesterone and DHP concentrations in the testes, indicating that *cyp17a1* regulates sex hormone production in fish [44]. In summary, these DEGs play an important role in the steroidogenesis of *M. terminalis*, and the species may share the same pattern of steroidogenesis with other teleost fish.

### 4.2. DEGs Involved in Gonadal Differentiation and Development

In mammals, gonadal differentiation and development are regulated by several genes and pathways, such as the WNT/β-catenin signaling pathway [45,46], the *sry*, *sox* [47], *dmrt*, and *fst* gene family. In vertebrates, gonadal differentiation is more intricate in fish compared to other species. Fish show several patterns of sexual differentiation, ranging from hermaphroditic to differentiated or undifferentiated gonochoristic species [48]. Among the genes identified in this study, several have been reported to be associated with gonadal differentiation in fish. In mandarin fish and Pengze crucian carp, *dmrt1* was found to be predominantly expressed in the testis [49], whereas in zebrafish ovary to testis transformation, *dmrt1* expression was found to be upregulated [50], suggesting that *dmrt1* mainly acts on testis development, which was similar to the result that the testis of M. terminalis had a higher *dmrt1* expression than that in the ovaries. Similarly, *dmrt2* is also highly expressed in the testis of *M. terminalis* and several other fish species, and its expression increases during testis development, suggesting that *dmrt2* may have similar roles as *dmrt1* [49,51]. *Fsta* is a follicular repressor-encoding gene, and treatment with follicular repressor reverses zebrafish females to males [52]. Thus, higher expression of *fst* in the testis of *M. terminalis* suggests that *fst* may also have a role in the development of its testis.

In addition, we identified several ovarian development-related genes. Foxl2 is a forkhead transcription factor essential for proper reproductive function in females. It is involved in almost all stages of ovarian development. A central role of FOXL2 is the lifetime maintenance of GC identity through the repression of testis-specific genes [53]. It has been reported to play a key role in ovarian differentiation in various fish species such as *Paralichthys olivaceus* [54,55]. The bone morphogenetic protein 15 (*bmp15*) and growth differentiation factor 9 (*gdf9*) genes are relevant members of the TGFβ superfamily that encode proteins secreted by the oocytes into the ovarian follicles [56]. In *M. terminalis*, *foxl2*, *bmp15*, and *gdf9* were highly expressed in the ovary, suggesting that they may play an important role in ovarian development in *M. terminalis*.

### 4.3. DEGs Involved in Gametogenesis and Gamete Maturation

In aquaculture, optimal gamete development is crucial for reproduction and enhanced yield of economically important fish. Meiosis in spermatocytes and oocytes is an essential step in gametogenesis. *cyb26a1* is identified as a gene that facilitates meiotic start in various fish species, and its downregulation accelerates the commencement of meiosis [57,58,59]. Additionally, both *sycp1* and *msh5* are essential for meiosis in mice. Mutations in *sycp1* result in meiotic arrest and spermatocyte mortality in males [60], whereas such mutations in female zebrafish cause a decrease in normal offspring, and mutations in males cause sterility [61]. Murine Msh5 facilitates the synapsis of homologous chromosomes during meiotic prophase I [62]. In *M. terminalis*, all three meiosis-associated genes are upregulated in the testis, potentially correlating with the increased incidence of meiosis in the spermatheca.

We have also found genes exclusive to spermatogenesis and oogenesis. In Nile tilapias, mutations in *rln3a* result in decreased sperm tail length and the formation of two-tailed spermatozoa [63], while *sox30*-deficient female mice exhibit normal phenotypes, and males are sterile [64]. In addition, TEKT4 protein is localized to the flagellum of mouse spermatozoa, and deletion of *tekt4* results in reduced fertility in male mice [65], suggesting that it is important for spermatogenesis. In addition, SYCP2L, which is important for meiosis in the oocyte, is a paralogue of the synaptonemal complex protein SYCP2 and is expressed exclusively in oocytes [66], and the gene *sycp2l* was highly expressed in the ovary of *M. terminalis.* These genes are differentially expressed in the gonads of *M. terminalis*, suggesting that they are important for spermatogenesis and oogenesis in *M. terminalis*.

## 5. Conclusions

We present the first transcriptome data from the gonads (testis and ovary) of *M. terminalis*, utilizing Illumina sequencing. A total of 14,972 DEGs were identified, with 11,928 exhibiting upregulation in the testis and 3044 showing downregulation. Furthermore, we found numerous DEGs linked to steroidogenesis, gonadal differentiation and development, and gametogenesis in mammals and teleost fish, indicating that these genes are likely to play significant roles in *M. terminalis*. The results will provide data to underpin further studies on gonadal differentiation in *M. terminalis*, as well as its reproduction and breeding.

## Figures and Tables

**Table 1:** Primers used for qRT-PCR.

Gene ID	Gene	Sequence (5′-3′)	Product Size (bp)
Unigene0048221	*β-actin*	F: ACCACGGCCGAAAGAGAAAT	97
		R: AAGATGAGGCAGCAGTTCCC	
Unigene0006630	*cyp27a1*	F: GATTGTTGTGCGTGCACTGT	135
		R: AGCGCTGAGGAATACGGATG	
Unigene0010781	*ch25h*	F: TGGGCTGCCACCCATTTAG	99
		R: GATGCAGTGCCCAAGGAAAG	
Unigene0002701	*cyp11a1*	F: GGTCTGTACGCTATGGGTCG	102
		R: CAGGCTCCTGAAGTAGTGGC	
Unigene0056686	*star*	F: TGGTTACACACGAGGTGTCG	115
		R: GCAGCTCCGCCCAAATAAAC	
Unigene0029504	*hsd17b12a*	F: GCTGGCGTTGTTTCTGTTGT	130
		R: TTTCCCAATCCCATCCGTGG	
Unigene0059133	*hsd17b1*	F: ACGGACGAATCCTTGTGACC	112
		R: ATAGCCAGGCTCTCACATGC	
Unigene0098050	*cyp11b*	F: AGGACTTCTGCCGTTCACTG	140
		R: TTCTCCGTACAGAACGTGGC	
Unigene0097311	*dmrt1*	F: AACCACGGATTCGTGTCTCC	106
		R: CCATGACCCGCTGTCTTTCA	
Unigene0055493	*dmrt2*	F: GCAGGTAGATGTGACCCACC	107
		R: CTGGAGCTTCATCCGTCCTC	
Unigene0061978	*foxl2*	F: ATAACTCGTGGTCTCCTGCG	126
		R: TCTGCACACGCGAATATGGG	
Unigene0081098	*cyp26a1*	F: AATTGGGCATCTACTCGCCT	142
		R: TCAAAGGTTTTGAGCGCCAC	
Unigene0045041	*sycp1*	F: GCTGAAGTCAACGCAAGCAA	128
		R: GAAACGGGTGTCCTCAAGGT	
Unigene0030921	*rln3*	F: ACCGAGAGACTTCGGAGTGA	124
		R: ATACGAGTCCAGGAGCCAGT	
Unigene0005965	*gdf9*	F: TGTACCTCAACGACACCAGC	146
		R: GGTGCCCTTTAGGGTTCCTC	
Unigene0002552	*bmp15*	F: TCACTGGATCATTGCACCCC	94
		R: GTGGTTGGGCGAATTGTAGC	
Unigene0089400	*zp3*	F: CAGTGGCAAAACTGACGTGG	95
		R: GTTGGGAGGGATAGGATGCG	

**Table 2:** Summary statistics of the gonadal RNA-seq data.

Sample	Reads Number	Total Base	Q20(%)	Q30(%)	GC Content(%)
ovary-1	53,679,572	7,989,217,225	99.26	97.29	46.52
ovary-2	44,297,662	6,605,257,416	99.27	97.35	45.90
ovary-3	43,255,124	6,449,759,681	99.27	97.34	46.73
testis-1	50,405,048	7,516,704,077	99.31	97.49	45.78
testis-2	38,698,706	5,779,517,823	99.26	97.31	45.81
testis-3	45,877,336	6,839,458,852	99.28	97.39	45.63
Mean	46,035,575	6,863,319,179	99.28	97.36	46.06
Total	276,213,448	41,179,915,074			

**Table 3:** Summary statistics of gonadal transcriptome assembly and annotation.

Type	Database	Number
Assembly	Gene number (#)	84,886
	Total length (nt)	92,262,782
	Average length (nt)	1086
	Max length (nt)	20,142
	Min length (nt)	201
	N50 (nt)	2238
	Total number of unigenes	42,322
	Unigenes match against Nr	40,529
Annotation	Unigenes match against SwissProt	25,877
	Unigenes match against KEGG	25,801
	Unigenes match against GO	25,055
	Unigenes match against KOG	20,139

**Table 4:** The patterns of DEGs related to reproduction, gonad development, and differentiation (Testis_vs_Ovary).

ID	log2(FC)	*p*-Value	FDR	Nr Annotation	Gene Name
Unigene0091813	1.644	3.31 × 10^−4^	3.05 × 10^−3^	adenylate cyclase type 5	*adcy5*
Unigene0050831	2.933	6.84 × 10^−7^	1.27 × 10^−5^	apolipoprotein Eb	*apoeb*
Unigene0081017	4.638	1.39 × 10^−4^	1.42 × 10^−3^	beta-carotene oxygenase1 like	*bco1*
Unigene0093028	7.066	1.15 × 10^−34^	6.04 × 10^−32^	beta-carotene oxygenase 2b	*bco2b*
Unigene0002552	−3.939	4.31 × 10^−20^	6.08 × 10^−18^	bone morphogenetic protein 15	*bmp15*
Unigene0032803	6.471	9.54 × 10^−16^	7.84 × 10^−14^	bone morphogenetic protein 8 A-like	*bmp8a*
Unigene0010781	2.219	5.12 × 10^−3^	3.17 × 10^−2^	cholesterol 25-hydroxylase-like protein	*ch25h*
Unigene0002701	−3.099	4.51 × 10^−6^	6.92 × 10^−5^	cholesterol side-chain cleavage enzyme, mitochondrial	*cyp11a1*
Unigene0098050	6.293	7.30 × 10^−14^	4.69 × 10^−12^	cytochrome P45011B, mitochondrial	*cyp11b*
Unigene0047166	−2.829	4.12 × 10^−6^	6.40 × 10^−5^	teroid 17-alpha-hydroxylase/17, 20lyase	*cyp17a1*
Unigene0004230	−5.369	1.48 × 10^−14^	1.04 × 10^−12^	Aromatase	*cyp19a1a*
Unigene0081098	−4.427	2.00 × 10^−31^	8.18 × 10^−29^	cytochrome P45026A1	*cyp26a1*
Unigene0061096	6.182	4.75 × 10^−6^	7.23 × 10^−5^	cytochrome P45026C1	*cyp26b1*
Unigene0006630	2.913	7.94 × 10^−5^	8.72 × 10^−4^	sterol 26-hydroxylase, mitochondrial	*cyp27a1*
Unigene0026663	2.404	5.64 × 10^−8^	1.32 × 10^−6^	daz-associated protein 1	*dazap1*
Unigene0052997	2.326	4.64 × 10^−6^	7.07 × 10^−5^	deleted in azoospermia-like	*dazl*
Unigene0059765	−1.266	3.88 × 10^−4^	3.50 × 10^−3^	putative ATP-dependent RNA helicase DDX5	*ddx5*
Unigene0055695	−2.318	3.91 × 10^−8^	9.44 × 10^−7^	probable ATP-dependent RNA helicase DDX52	*ddx52*
Unigene0097311	7.810	1.39 × 10^−48^	1.74 × 10^−45^	doublesex-and mab-3-related transcription factor 1	*dmrt1*
Unigene0055493	2.063	7.38 × 10^−3^	4.29 × 10^−2^	doublesex-and mab-3-related transcription factor 2b	*dmrt2*
Unigene0010711	6.154	1.57 × 10^−7^	3.36 × 10^−6^	doublesex-and mab-3-related transcription factor 3a	*dmrt3a*
Unigene0090348	−3.991	3.16 × 10^−11^	1.34 × 10^−9^	estrogen receptor	*esr1*
Unigene0083632	5.411	1.27 × 10^−4^	1.31 × 10^−3^	fibroblast growth factor 10b	*fgf10*
Unigene0097340	3.638	2.46 × 10^−9^	7.47 × 10^−8^	fibroblast growth factor 12	*fgf12*
Unigene0053217	2.282	3.88 × 10^−3^	2.51 × 10^−2^	fibroblast growth factor 13	*fgf13*
Unigene0054944	4.554	1.83 × 10^−13^	1.11 × 10^−11^	fibroblast growth factor 14	*fgf14*
Unigene0045081	3.597	4.88 × 10^−4^	4.26 × 10^−3^	fibroblast growth factor 18a	*fgf18*
Unigene0020029	6.452	8.78 × 10^−5^	9.51 × 10^−4^	fibroblast growth factor 20a	*fgf20*
Unigene0012888	7.288	3.04 × 10^−6^	4.87 × 10^−5^	fibroblast growth factor 5	*fgf5*
Unigene0015061	4.718	2.15 × 10^−4^	2.09 × 10^−3^	fibroblast growth factor 7	*fgf7*
Unigene0056515	3.222	7.83 × 10^−6^	1.13 × 10^−4^	fibroblast growth factor 8	*fgf8a*
Unigene0043775	9.758	2.25 × 10^−4^	2.17 × 10^−3^	fibroblast growth factor 8b	*fgf8b*
Unigene0061978	−5.061	4.58 × 10^−11^	1.89 × 10^−9^	forkhead box protein L2a	*foxl2*
Unigene0067956	3.596	1.22 × 10^−3^	9.36 × 10^−3^	Follistatin a	*fsta*
Unigene0005965	−3.924	6.18 × 10^−19^	7.55 × 10^−17^	growth/differentiation factor 9	*gdf9*
Unigene0036772	2.372	4.96 × 10^−6^	7.51 × 10^−5^	3-hydroxy-3-methylglutaryl-CoA reductase-A	*hmgcr*
Unigene0059133	−4.930	4.35 × 10^−24^	9.78 × 10^−22^	estradiol 17-beta-dehydrogenase 1	*hsd17b1*
Unigene0029504	−4.299	4.34 × 10^−28^	1.35 × 10^−25^	very-long-chain 3-oxoacyl-CoA reductase-A	*hsd17b12a*
Unigene0078112	2.075	3.52 × 10^−3^	2.32 × 10^−2^	branched-chain-amino-acid aminotransferase, mitochondrial	*hsd17b14*
Unigene0094765	−2.010	7.06 × 10^−7^	1.31 × 10^−5^	low-density lipoprotein receptor adapter protein 1a	*ldlrap1*
Unigene0089025	8.417	2.24 × 10^−34^	1.15 × 10^−31^	mutS protein homolog 5	*msh5*
Unigene0090761	2.502	1.61 × 10^−4^	1.62 × 10^−3^	nuclear receptor subfamily 1, group D, member 4a	*nr1d4a*
Unigene0091665	2.127	5.11 × 10^−7^	9.77 × 10^−6^	glucocorticoid receptor	*nr3c1*
Unigene0076031	1.698	2.07 × 10^−3^	1.48 × 10^−2^	mineralocorticoid receptor	*nr3c2*
Unigene0045148	4.184	1.23 × 10^−7^	2.70 × 10^−6^	proprotein convertase subtilisin/kexintype5	*pcsk5*
Unigene0030921	4.250	4.48 × 10^−6^	6.88 × 10^−5^	prorelaxin H1	*rln3*
Unigene0003816	1.493	4.63 × 10^−3^	2.91 × 10^−2^	retinoid x receptor, beta	*rxrba*
Unigene0057144	2.052	1.87 × 10^−3^	1.35 × 10^−2^	retinoic acid receptor RXR-beta-B	*rxrbb*
Unigene0060296	5.630	7.98 × 10^−27^	2.23 × 10^−24^	transcription factor SOX-30-like	*sox30*
Unigene0056686	−3.370	2.19 × 10^−7^	4.56 × 10^−6^	steroidogenic acute regulatory protein, mitochondrial	*star*
Unigene0045041	4.845	2.12 × 10^−13^	1.27 × 10^−11^	synaptonemal complex protein 1	*sycp1*
Unigene0013279	−7.954	3.33 × 10^−60^	7.20 × 10^−57^	synaptonemal complex protein 2-like	*sycp2l*
Unigene0034578	10.693	2.07 × 10^−7^	4.32 × 10^−6^	tektin-4	*tekt4*
Unigene0008414	2.868	7.65 × 10^−5^	8.44 × 10^−4^	transforming growth factor beta-2 proprotein	*tgf-β2*
Unigene0068547	−1.109	3.88 × 10^−3^	2.51 × 10^−2^	transforming growth factor beta receptor-associated protein 1 homolog	*tgf-βrap1*
Unigene0089400	−8.467	4.99 × 10^−73^	2.22 × 10^−69^	zona pellucida sperm-binding protein 3-like	*zp3*
Unigene0017634	−8.647	8.75 × 10^−47^	9.72 × 10^−44^	zona pellucida sperm-binding protein 4-like	*zp4*

## Data Availability

The data presented in this study are openly available in NCBI/Bioproject, reference number PRJNA1278959.

## Supplementary Materials

The following supporting information can be downloaded at: https://www.mdpi.com/article/10.3390/ani15152184/s1, Table S1. Top 50 terms of GO (Testis_vs_Ovary). Table S2. Top 50 pathway of KEGG (Testis_vs_Ovary).
