# Supplementary material for: De Novo Assembly, Characterization and Comparative Transcriptome Analysis of the Mature Gonads in Megalobrama terminalis
Source: Animals (Basel). 2025 Jul 24;15(15):2184. doi: 10.3390/ani15152184 (PMC12345467; doi:10.3390/ani15152184)
Supplement: Supplementary file 1 [file animals-15-02184-s001.zip › animals-3738415-supplementary.pdf]

animals

MDPI

Supplementary Table S1. Top 50 terms of GO (Testis_vs_Ovary).						
**GO ID**	**class**	**Descrption**	**Pvalue**	**Qvalue**	**Down**	**Up**
GO:0065007	Biological Process	biological regulation	7.10E-40	9.49E-36	2944	965
GO:0050789	Biological Process	regulation of biological process	5.32E-36	3.56E-32	2781	918
GO:0050794	Biological Process	regulation of cellular process	5.87E-34	2.61E-30	2663	890
GO:0005515	Molecular Function	protein binding	7.59E-32	2.63E-28	2765	773
GO:0032501	Biological Process	multicellular organismal process	2.80E-30	9.36E-27	1819	561
GO:0016043	Biological Process	cellular component organization	5.39E-28	1.44E-24	1857	608
GO:0071840	Biological Process	cellular component organization or biogenesis	2.38E-27	5.30E-24	1951	664
GO:0032502	Biological Process	developmental process	1.04E-25	1.98E-22	1690	557
GO:0048856	Biological Process	anatomical structure development	3.77E-24	6.30E-21	1576	514
GO:0007275	Biological Process	multicellular organism development	2.93E-22	4.35E-19	1462	475
GO:0048731	Biological Process	system development	1.47E-21	1.97E-18	1274	417
GO:0006996	Biological Process	organelle organization	2.44E-20	2.97E-17	1227	398
GO:0044782	Biological Process	cilium organization	1.84E-19	2.05E-16	167	18
GO:0060271	Biological Process	cilium assembly	8.44E-19	8.68E-16	158	17

GO:0007154	Biological Process	cell communication	9.59E-18	9.15E-15	1624	526
GO:0120036	Biological Process	plasma membrane bounded cell projection organization	5.44E-17	4.85E-14	530	113
GO:0120031	Biological Process	plasma membrane bounded cell projection assembly	4.50E-16	3.76E-13	209	27
GO:0023052	Biological Process	signaling	5.85E-16	4.59E-13	1578	515
GO:0030154	Biological Process	cell differentiation	1.92E-15	1.35E-12	1086	338
GO:0048869	Biological Process	cellular developmental process	1.92E-15	1.35E-12	1105	347
GO:0019222	Biological Process	regulation of metabolic process	2.54E-15	1.70E-12	1597	575
GO:0050896	Biological Process	response to stimulus	1.18E-14	7.53E-12	2284	769
GO:0031323	Biological Process	regulation of cellular metabolic process	1.62E-14	9.74E-12	1463	537
GO:0007165	Biological Process	signal transduction	1.68E-14	9.74E-12	1451	483
GO:0060255	Biological Process	regulation of macromolecule metabolic process	1.75E-14	9.74E-12	1514	543
GO:0120025	Cellular Component	plasma membrane bounded cell projection	6.16E-15	1.02E-11	670	142
GO:0051716	Biological Process	cellular response to stimulus	2.00E-14	1.03E-11	1882	647
GO:0030030	Biological Process	cell projection organization	2.01E-14	1.03E-11	545	119
GO:0048519	Biological Process	negative regulation of biological process	3.61E-14	1.79E-11	1193	439
GO:0007017	Biological Process	microtubule-based process	5.30E-14	2.53E-11	320	78
GO:0080090	Biological Process	regulation of primary metabolic process	5.66E-14	2.61E-11	1431	524

GO:0043227	Cellular Component	membrane-bounded organelle	3.45E-14	2.86E-11	2453	940
GO:0048513	Biological Process	animal organ development	1.48E-13	6.57E-11	947	312
GO:0051171	Biological Process	regulation of nitrogen compound metabolic process	1.70E-13	7.31E-11	1400	512
GO:0051276	Biological Process	chromosome organization	1.96E-13	8.20E-11	574	182
GO:0030031	Biological Process	cell projection assembly	2.56E-13	1.04E-10	217	30
GO:0006464	Biological Process	cellular protein modification process	4.59E-13	1.80E-10	1112	363
GO:0048523	Biological Process	negative regulation of cellular process	5.35E-13	2.04E-10	1068	407
GO:0048518	Biological Process	positive regulation of biological process	5.96E-13	2.21E-10	1256	446
GO:0009893	Biological Process	positive regulation of metabolic process	1.22E-12	4.39E-10	798	302
GO:0048468	Biological Process	cell development	1.70E-12	5.98E-10	717	221
GO:0036211	Biological Process	protein modification process	2.19E-12	7.51E-10	1136	383
GO:0042995	Cellular Component	cell projection	1.70E-12	9.42E-10	682	145
GO:0003008	Biological Process	system process	3.19E-12	1.07E-09	515	135
GO:0048522	Biological Process	positive regulation of cellular process	3.57E-12	1.16E-09	1175	412
GO:0007399	Biological Process	nervous system development	5.26E-12	1.67E-09	757	216
GO:0010604	Biological Process	positive regulation of macromolecule metabolic process	1.59E-11	4.95E-09	749	287
GO:0005488	Molecular Function	binding	4.05E-12	7.01E-09	4589	1479

GO:0042221	Biological Process	response to chemical	4.54E-11	1.38E-08	1007	357
GO:0031325	Biological Process	positive regulation of cellular metabolic process	4.81E-11	1.43E-08	726	267

Supplementary Table S2. Top 50 pathway of KEGG (Testis_vs_Ovary).

**KEGG_A_class**	**KEGG_B_class**	**Pathway**	**Count (14490)**	**Pathway ID**
Metabolism	Global and overview maps	Metabolic pathways	2979	ko01100
Human Diseases	Neurodegenerative disease	Pathways of neurodegeneration - multiple diseases	1116	ko05022
Human Diseases	Cancer: overview	Pathways in cancer	963	ko05200
Human Diseases	Infectious disease: viral	Herpes simplex virus 1 infection	881	ko05168
Human Diseases	Neurodegenerative disease	Alzheimer disease	806	ko05010
Human Diseases	Neurodegenerative disease	Amyotrophic lateral sclerosis	769	ko05014
Environmental Information Processing	Signaling molecules and interaction	Neuroactive ligand-receptor interaction	734	ko04080
Human Diseases	Infectious disease: viral	Human papillomavirus infection	706	ko05165
Environmental Information Processing	Signal transduction	Calcium signaling pathway	694	ko04020
Environmental Information Processing	Signal transduction	PI3K-Akt signaling pathway	692	ko04151
Human Diseases	Neurodegenerative disease	Prion disease	676	ko05020
Cellular Processes	Cell motility	Cytoskeleton in muscle cells	662	ko04820
Human Diseases	Infectious disease: viral	Coronavirus disease - COVID-19	662	ko05171
Environmental Information Processing	Signal transduction	MAPK signaling pathway	654	ko04010

Human Diseases	Neurodegenerative disease	Huntington disease	647	ko05016
Human Diseases	Infectious disease: bacterial	Salmonella infection	591	ko05132
Human Diseases	Neurodegenerative disease	Parkinson disease	589	ko05012
Cellular Processes	Transport and catabolism	Endocytosis	581	ko04144
Cellular Processes	Transport and catabolism	Phagosome	567	ko04145
Human Diseases	Infectious disease: bacterial	Shigellosis	563	ko05131
Environmental Information Processing	Signal transduction	cAMP signaling pathway	559	ko04024
Environmental Information Processing	Signaling molecules and interaction	Cell adhesion molecules	551	ko04514
Human Diseases	Infectious disease: viral	Human T-cell leukemia virus 1 infection	530	ko05166
Human Diseases	Infectious disease: viral	Human cytomegalovirus infection	516	ko05163
Human Diseases	Infectious disease: viral	Human immunodeficiency virus 1 infection	511	ko05170
Human Diseases	Cardiovascular disease	Lipid and atherosclerosis	497	ko05417
Human Diseases	Infectious disease: viral	Epstein-Barr virus infection	490	ko05169
Human Diseases	Infectious disease: bacterial	Pathogenic Escherichia coli infection	489	ko05130
Cellular Processes	Cell motility	Motor proteins	479	ko04814
Human Diseases	Infectious disease: bacterial	Tuberculosis	469	ko05152
Human Diseases	Cancer: overview	Viral carcinogenesis	445	ko05203
Human Diseases	Infectious disease: viral	Kaposi sarcoma-associated herpesvirus infection	437	ko05167
Environmental Information Processing	Signal transduction	Rap1 signaling pathway	435	ko04015
Cellular Processes	Cell motility	Regulation of actin cytoskeleton	433	ko04810
Human Diseases	Cancer: overview	Proteoglycans in cancer	432	ko05205
Cellular Processes	Cellular community - eukaryotes	Focal adhesion	430	ko04510

Organismal Systems	Environmental adaptation	Thermogenesis	426	ko04714
Organismal Systems	Circulatory system	Adrenergic signaling in cardiomyocytes	423	ko04261
Organismal Systems	Endocrine system	Oxytocin signaling pathway	423	ko04921
Human Diseases	Cancer: overview	Chemical carcinogenesis - reactive oxygen species	422	ko05208
Human Diseases	Cancer: overview	Chemical carcinogenesis - receptor activation	420	ko05207
Genetic Information Processing	Translation	Ribosome	419	ko03010
Environmental Information Processing	Signal transduction	cGMP-PKG signaling pathway	416	ko04022
Human Diseases	Cardiovascular disease	Diabetic cardiomyopathy	410	ko05415
Genetic Information Processing	Folding, sorting and degradation	Protein processing in endoplasmic reticulum	406	ko04141
Cellular Processes	Cell growth and death	Cellular senescence	397	ko04218
Organismal Systems	Immune system	NOD-like receptor signaling pathway	393	ko04621
Environmental Information Processing	Signal transduction	Ras signaling pathway	381	ko04014
Human Diseases	Cancer: overview	Transcriptional misregulation in cancer	380	ko05202
